# Supplementary material for: How the AHR Became Important in Cancer: The Role of Chronically Active AHR in Cancer Aggression
Source: Int J Mol Sci. 2020 Dec 31;22(1):387. doi: 10.3390/ijms22010387 (PMC7795223; doi:10.3390/ijms22010387)
Supplement: Supplementary file 1 [file ijms-22-00387-s001.pdf]

Gene (1-99)	Adjusted p value	Gene (100-199)	Adjusted p value	Gene (200-299)	Adjusted p value	Gene (300-399)
CYP1B1	8.69E-09	ST6GALNAC2	0.00175734	GBP4	0.007326569	TNFRSF11B
PLAT	1.87E-07	CPE	0.00175734	RAB18	0.007350148	MDGA1
PTGS2	1.22E-06	SPDEF	0.00175734	ABCG2	0.00735399	ERAP1
GALNT12	1.22E-06	ADAMTS9	0.001872896	LGR4	0.007391048	IRAK2
SERPINB2	1.68E-06	NCOA3	0.001895845	CENPI	0.007417989	EML5
IL1A	2.23E-06	MMP14	0.001919555	GRN	0.007437117	RASD1
PTPRJ	3.04E-06	EMP1	0.002000424	F2RL1	0.00777617	HPGD
SLC9A3R2	3.04E-06	MB21D2	0.002027358	OSR1	0.00777617	USO1
TXNIP	5.78E-06	BTBD9	0.00213502	GBP2	0.007870641	FHL2
IER3	9.72E-06	EDEM2	0.002156054	ADAM8	0.007911032	BIRC3
PTPN22	1.80E-05	SLC7A11	0.002287646	PFKP	0.007957492	PLB1
TMEM45B	1.87E-05	IGFBP6	0.002287646	ELK3	0.008002603	FAM84B
MFI2	1.87E-05	FAH	0.00230407	SUSD1	0.008114623	SLC20A1
PLEKHF1	2.10E-05	RPP38	0.00230407	ABHD4	0.008227515	DLK2
SHISA2	2.10E-05	SHC4	0.002395643	IRF1	0.008358042	NFE2L3
SLC37A2	2.52E-05	TMEM156	0.002447991	MYD88	0.008408773	ARRDC4
TGFBR2	2.57E-05	IL24	0.002556957	DCLRE1C	0.0084807	MOB1B
FUCA1	2.97E-05	ARHGAP12	0.002560952	CFH	0.008525449	PVRL3
ADIRF	3.28E-05	ENPP1	0.002567723	USP6NL	0.008570325	SAMD4A
MAP4K4	3.61E-05	CYP1A1	0.002592256	GLIPR1	0.008705957	PGBD1
LAPTM5	3.62E-05	C10orf25	0.002603735	C1GALT1C1	0.008705957	TNFRSF21
TAP1	3.94E-05	SLC9A7	0.002648998	FOSL2	0.008724436	P4HA1
PDCD1LG2	4.11E-05	LINC01269	0.002741978	CAT	0.009018494	DUSP22
PDLIM5	8.87E-05	REPS2	0.002792547	GALNT6	0.00918253	SUPT7L
FNDC4	8.87E-05	TMEM158	0.002898532	C14orf169	0.009227068	C11orf74
EREG	9.55E-05	RIPK4	0.002898532	TPMT	0.009281091	DHRS3
TRAC	0.00010175	KCTD20	0.002909869	CMTM3	0.009659044	DDX58
EHF	0.000106252	LIPH	0.003008815	FHOD3	0.009712346	FUT3
PRRG4	0.000135466	DUSP6	0.003008815	MMP1	0.009943943	CLIP4
TIPARP	0.000135534	IL6	0.003008815	ZBED5-AS1	0.009943943	ANGPTL2
CXCL8	0.000160057	SYNJ2	0.003008815	CCNE1	0.010052928	CDC42EP2
NRP1	0.000160057	ASAP2	0.003008815	RAB23	0.010052928	KIN
CTSS	0.000165897	GATA3	0.003008815	C10orf55	0.010061291	MAB21L1
HPCAL1	0.000173119	ADPRH	0.003008815	PLAU	0.010061291	WBP5
GBP1	0.000180456	IL27RA	0.003008815	SMAGP	0.010061291	TGFB1I1
ERRFI1	0.000181298	NFE2L2	0.003008815	PSORS1C1	0.010061291	CMAHP
TNIK	0.000181298	AKAP5	0.003097639	CCNDBP1	0.010061291	FAM214B
LURAP1L	0.000191348	ACTBL2	0.003170298	TM7SF2	0.010080844	ANGPTL4
ICAM1	0.000191732	SERPINB9	0.003230449	LINC00472	0.010149621	FHDC1
ITGA2	0.000234191	SLC2A1	0.003242612	TRBV7-3	0.01035748	TSPAN13
MORC4	0.000257513	NFKBIE	0.003477046	C4orf19	0.010410563	NPNT
SPRED1	0.000294359	ADGRF1	0.003537329	UGDH	0.010540251	LINC01605
ZNF224	0.000338867	PITPNC1	0.003537329	ANKRD29	0.010629705	VPS11
SORL1	0.000353134	C15orf39	0.003542188	ELOVL6	0.010712793	ZSCAN16
UGCG	0.000365119	CREG1	0.003549755	PLPP1	0.010715669	ANKRD42

TACSTD2	0.000387554	TGFA	0.003549755	ALKBH1	0.010982774	GPR137
PLAUR	0.000387554	SPRY1	0.003553257	TMEM25	0.010982774	CDC42EP1
ARL4C	0.000387554	GYLTL1B	0.003553741	RNF167	0.01125514	SELL
ADGRF4	0.000407497	BRPF3	0.003573157	LINC00920	0.011389883	C19orf33
FAM69A	0.000437762	SPRY4	0.003723628	DTNA	0.011523819	SEC61G
HLA-E	0.000437762	CD82	0.00375608	RNF8	0.011533957	ARMCX6
C11orf24	0.000437762	NAAA	0.003779846	NGFRAP1	0.011653022	FAM102A
ANKRD37	0.000469335	ADORA2B	0.003802871	PPP1R18	0.011795165	KRT23
ARHGAP18	0.000475876	ENTPD3	0.003802871	NMNAT2	0.011889832	ETV6
SLC4A4	0.000513282	MAML2	0.003806164	SDC4	0.011889832	ZNF350
AHR	0.000525278	MOB3A	0.003860904	CD2AP	0.011889832	ZNF570
GBP3	0.000525278	GSTM3	0.003966613	PLEKHH2	0.011903464	HLA-DRB1
SMPDL3B	0.000525278	MID2	0.004146738	PIP4K2A	0.011919537	RNF114
CCNJL	0.000636608	TAF8	0.004185897	PSMB8	0.011923176	FTH1
PRKCA	0.00064348	HLA-C	0.004192003	GPR39	0.012245033	STAM2
ATP6V0D2	0.000711538	TMCC3	0.004236238	WDR37	0.012245894	ZNF264
GALNT3	0.000792901	PTPN21	0.004431991	PYCARD	0.012389506	SAYSD1
SHISA4	0.000810548	KDM6B	0.00445608	B4GALT6	0.012499147	JUN
SECTM1	0.000826364	RNU6-1161P	0.004478058	CLK1	0.012556894	ALOX5AP
HID1	0.000844166	FHL3	0.004709089	VAT1	0.012610294	HBEGF
SLC17A5	0.000844166	SH3PXD2A	0.004713704	ZFPL1	0.012647405	USP30-AS1
C15orf48	0.000849595	FAM174B	0.004929318	ACER3	0.012647405	OVOL1
CXCL11	0.000854451	SLC25A20	0.00504111	NLRP3	0.012841728	HYAL3
PSMB9	0.000873697	SIGIRR	0.005063829	SLC16A13	0.013074443	LIPG
TFAP2A-AS1	0.000873697	TSKU	0.005100798	MIR22HG	0.013074443	PLXNA3
KCNK6	0.000873697	BNIP3	0.00528604	IL15RA	0.013074443	MICB
CDKN1A	0.000892315	MYO5B	0.00541096	SMURF2	0.01313579	GULP1
TNIP1	0.000931776	SLC39A7	0.005591767	P2RY6	0.013212965	MIR222HG
JARID2	0.001033824	GATA6	0.005591903	ZNF567	0.013592923	BRE
OPTN	0.001095746	PLD5	0.005637688	ANTXR2	0.013608878	PPFIBP1
TMEM63A	0.001164648	SEC14L2	0.005637688	CARD6	0.013622318	TGM2
KRTAP2-3	0.001164648	PTK6	0.005637688	CPA4	0.013634445	TMEM106A
C6orf226	0.001164648	SIK3	0.005868849	TFPT	0.013691185	FAM210B
SMARCA1	0.001173226	SLC4A11	0.005868849	FAM105A	0.013732542	FAM127B
DDR1	0.001187836	WFS1	0.005924711	BCAM	0.013843431	ATP5L
ZC3H12C	0.001213083	SLC29A1	0.005924711	CTSLP2	0.01393392	ARMCX3
IL31RA	0.001213083	F8A1	0.005924711	SLCO4A1	0.014367986	LINC01424
TRIOBP	0.001277321	RRP36	0.005924711	G0S2	0.0144328	CHRDL1
GBP6	0.001329045	IL22RA1	0.005980089	TCTA	0.014613189	SLCO3A1
MANSC1	0.001329045	CAPN1	0.006001289	NHS	0.014613189	ZEB1
SLC30A4	0.001340209	C11orf68	0.006025083	GPAT3	0.014977651	ZNF879
CSF2	0.00144202	NKX3-1	0.006060099	MAX	0.014999237	BTN2A1
ROR1	0.001500586	TESK1	0.006223273	SUCO	0.015064211	TRIM16L
ENPP4	0.001564466	FAM127A	0.006357988	GNL1	0.015069456	C11orf63
TMEM171	0.001564466	OTUB2	0.00638646	SLC6A14	0.015251851	SVIL-AS1
LAMP3	0.00158998	INPPL1	0.00638646	APAF1	0.015350774	SRPK1
ATP9A	0.001630765	HAS3	0.006426413	GRAMD3	0.015350774	SLC9A7P1

DNMBP	0.00163213	C6orf136	0.006587402	PSMD6-AS2	0.015490806	GMPPA
ERN1	0.001656095	MREG	0.00671173	APOBEC3D	0.015490806	CNKSR2
LPCAT2	0.001679827	ICAM2	0.006749661	TCEAL1	0.015490806	IL23A
TTC30A	0.001706602	SRPX2	0.006841876	SH3D21	0.015490806	ISM1
PTGES	0.001706602	FOXF2	0.007058985	HLA-K	0.015495033	ZFAND2A
CST7	0.001706602	CLMP	0.007154586	FMNL3	0.015626705	LAMC2
HAS2	0.001721338	REP15	0.007174677	LPIN2	0.015686191	HMGA1
				ZNF134	0.016127259	AKAP12

Adjusted p value	Gene (400-499)	Adjusted p value	Gene (500-599)	Adjusted p value	Gene (600-644)	Adjusted p value
0.016156571	MAP3K8	0.023375319	STRN	0.033941866	POLB	0.045379834
0.016343572	ADAM28	0.023637744	S100A7	0.034119855	FAM222A-AS1	0.04555556
0.016369458	ACOXL	0.023680659	SULT2B1	0.034119855	PIP4K2C	0.04555556
0.016438854	CST4	0.023680659	HOXC13	0.034310424	CEP250	0.045569429
0.016509469	GDA	0.023682367	HCCS	0.034349371	CRYGC	0.045569429
0.016679626	PXYLP1	0.023724662	FBXO3	0.034435513	ADNP-AS1	0.045703009
0.01685363	BICC1	0.024547619	TUBGCP2	0.034596288	DLG3	0.045800647
0.017064984	DNAJC1	0.024547619	CTNS	0.03470494	ANKRD30BL	0.045947094
0.017095889	BTN2A2	0.024927732	EEF1A1	0.03470494	LINC00702	0.04596246
0.017160776	SNRPA1	0.024927732	FZD7	0.034877763	RNF141	0.045978045
0.01750548	PXDN	0.024967642	FMNL1	0.035002307	NLK	0.046278061
0.017579629	TOR1B	0.024967642	CXCL16	0.035115054	CCND1	0.046556206
0.017854546	KPNA7	0.025182137	RNU6ATAC11P	0.035165463	CDCP1	0.046687602
0.017908518	TFF1	0.025182137	TRPT1	0.035475854	PUS3	0.046691115
0.017908518	DNAJA4	0.025446272	ZDHHC9	0.035576786	DYRK1B	0.046691115
0.017908518	ADAM19	0.025514925	TMOD1	0.035784408	TPM2	0.04681103
0.017908518	MYL9	0.025514925	RGS10	0.035885082	ASB1	0.04682624
0.017908518	AUH	0.025516031	FRMD6	0.035937342	PPP1R14C	0.046916108
0.017908518	LINC01191	0.025539262	VARS2	0.036205814	RIC1	0.046916108
0.017908518	LIF	0.025670833	GPRC5C	0.036256956	MRPL33	0.046916108
0.017908518	GPR68	0.025670833	IL4R	0.036277	PNPLA3	0.046928887
0.017908518	PLS1	0.025856315	TMEM164	0.036438886	TTC30B	0.047095923
0.017908518	PBX2	0.025910042	NAV1	0.036487135	RTN2	0.047304061
0.017908518	BCORL1	0.026112305	FBXW10	0.036487135	PRSS22	0.047527694
0.018092329	MECOM	0.026188128	IKZF2	0.036915863	SMURF1	0.047581021
0.018097851	LINC00665	0.026245761	LINC00857	0.036915863	TIMP1	0.047826943
0.018103082	VSIG1	0.026425422	GNG5	0.036915863	SAMD12	0.047826943
0.018356674	ZNF112	0.026425422	SNPH	0.037537558	NEIL1	0.047856187
0.018367811	DENND2D	0.026425422	NIPAL1	0.037686487	AGPAT3	0.048009793
0.018367811	SPINT2	0.026425422	DDAH2	0.037884283	RPS26	0.048111441
0.018550593	ATP8A2	0.026425422	FN3K	0.037971042	AQP11	0.048340467
0.018637826	PHF11	0.026682351	MMP19	0.038005073	COMMD9	0.048372744
0.018687224	TTC9	0.026762794	FOXA1	0.038326488	SLC35A3	0.048431338
0.018942254	PQLC3	0.026937467	ALDH3A2	0.038330231	PTPN12	0.048576098
0.018969963	FLG	0.026962409	PLEKHG4B	0.03856115	UBA6-AS1	0.048576098
0.018969963	WDR44	0.026965209	ZNF185	0.038700803	SERPIND1	0.048576098
0.019038551	LPAR1	0.026965209	HIST1H2AG	0.038802046	RBPJ	0.048589109
0.019085658	ALDH1A3	0.027096866	AMPD3	0.03880321	MARCKSL1	0.048806184
0.019085658	SYTL4	0.027127696	PTHLH	0.039016166	FAM19A3	0.048826659
0.019240502	DAAM1	0.027198621	ARCN1	0.039157611	NRIP3	0.04902509
0.019341426	GCNT1	0.027202188	FBXW7	0.039199556	ZNF550	0.049132111
0.019348237	CMTR1	0.027345487	DLG4	0.039252826	PRPF4B	0.049433006
0.019463698	PDP1	0.027442638	HAP1	0.039753237	AXIN2	0.049477432
0.019475215	HLA-DRA	0.027943897	KIF3B	0.039903688	TMC5	0.04981934
0.019475215	ZNF808	0.028054053	AFAP1	0.040013434	LRRC1	0.04981934

0.019475215	SLC25A30	0.028152061	ZFAND3	0.040539522	MARVELD2	0.049942672
0.019541374	NQO1	0.02819653	ADAMTS1	0.040548934		
0.019541374	OCRL	0.028220205	LINC01468	0.040565076		
0.019615309	MRPL14	0.028220205	TGFB1	0.040565076		
0.019642967	SYTL2	0.028280984	SENCR	0.040635943		
0.019644058	IKBKE	0.028635443	DZANK1	0.040654901		
0.019644058	OARD1	0.028687644	DGKE	0.040662809		
0.019683334	CALB2	0.029049312	ERO1B	0.040937189		
0.019797507	FAM3C	0.029062067	PARD3	0.040954034		
0.019917398	PRKCD	0.029076941	ELF3	0.042127516		
0.02004728	SMIM13	0.029546234	SIPA1	0.042127516		
0.02004728	RNF128	0.029733051	MT1L	0.042130351		
0.02004728	FBXO18	0.029741987	FOXQ1	0.042271641		
0.02004728	SLC43A3	0.029795551	GALM	0.042283951		
0.02004728	GNG11	0.029919315	PANX1	0.042348011		
0.02004728	MAST4-AS1	0.029919315	TLR2	0.042460427		
0.020101972	SH3TC2	0.030092854	IFITM10	0.042473707		
0.020161544	MAFG	0.030328693	CD274	0.042473707		
0.020248687	LINC00518	0.030328693	TRAF6	0.042642995		
0.020272917	ANKRD13A	0.030462386	IRF6	0.042724516		
0.020277818	PLBD2	0.030559813	CD46	0.042780005		
0.020277818	KRT15	0.030636424	ANKLE1	0.042866451		
0.020348665	OSTF1	0.030636424	NIPA1	0.042969576		
0.020433898	RSU1	0.030636424	VGLL1	0.043059771		
0.020889285	INSIG2	0.030785223	ADAMTS6	0.043059771		
0.020889285	KRT10	0.030785223	HLA-F	0.043187635		
0.020889887	GGT5	0.031246681	ELF4	0.043187635		
0.021052744	TSR2	0.031284631	CYP4F3	0.043187635		
0.021052744	FOSL1	0.031286867	ZNF416	0.043187635		
0.021461759	CCND3	0.031334088	PPP1R15A	0.043279808		
0.021461759	PARD6B	0.031629937	KIAA1217	0.043286327		
0.021461759	ZSCAN9	0.031760459	CREG2	0.043287953		
0.021464187	BCL2L1	0.032212053	KMO	0.043361413		
0.021470443	RPL7L1	0.032487029	STAP2	0.043361413		
0.021545624	TRIM8	0.032620996	NFYA	0.043361413		
0.021545624	CHST14	0.032620996	FER1L4	0.043361413		
0.021545624	SAMD15	0.032682163	HLA-G	0.043389556		
0.021853842	CRAT	0.032776072	MID1IP1-AS1	0.043440707		
0.021853842	TBRG1	0.032776072	TMEM263	0.043467151		
0.021931395	SORT1	0.032793151	IFT46	0.043581893		
0.021931395	IL1R2	0.032793151	CTC1	0.044065786		
0.021983553	G3BP2	0.032793151	EPGN	0.044065786		
0.022300907	CLDN23	0.032793151	HCAR1	0.044065786		
0.022300907	BAD	0.032804124	CHRNB1	0.044126916		
0.022329893	UPF2	0.03296191	STK40	0.044140233		
0.022329893	KCNRG	0.03296191	PLAC8	0.044187049		
0.022700658	PRDM8	0.033213916	LENG8	0.044187049		

0.022700658	ANKRD22	0.033431339	ZBTB4	0.04428393
0.023150655	LCMT1	0.033554546	DHRS9	0.044327232
0.02316008	STRA6	0.033560206	NRGN	0.044355107
0.023225537	FBLN7	0.033560206	GDE1	0.044394548
0.02328184	FAM126B	0.033652361	BRD2	0.044394548
0.023325015	YIPF4	0.033779048	CUL2	0.044762166
0.023325015	TNFAIP2	0.033796265	MT-TP	0.045137719
0.023325015	ABTB2	0.033818495	PPP1CB	0.045302093
